# Structure, regulatory factors and cancer-related physiological effects of ADAM9

**DOI:** 10.1080/19336918.2020.1817251

**Published:** 2020-09-12

**Authors:** MA Haoyuan, LI Yanshu

**Affiliations:** a Department of Clinical Medicine, China Medical University, Liaoning, Shenyang, China; b Department of Cell Biology, Key Laboratory of Cell Biology, Ministry of Public Health, and Key Laboratory of Medical Cell Biology, Ministry of Education, China Medical University, Liaoning, Shenyang, China

**Keywords:** ADAMs, ADAM9, miRNAs, metalloproteinase, malignant tumor, tumor invasion

## Abstract

The ADAMs family belongs to the transmembrane protein superfamily of zinc-dependent metalloproteases, which consists of multiple domains. These domains have independent but complementary functions that enable them to participate in multiple biological processes. Among them, ADAM9 can not only participate in the degradation of extracellular matrix as a metalloprotease, but also mediate tumor cell adhesion through its deintegrin domain, which is closely related to tumor invasion and metastasis. It is widely expressed in a variety of tumor cells and can affect the proliferation, invasion and metastasis of related cancer cells. We provide our views on current progress, its increasing importance as a strategic treatment goal, and our vision for the future of ADAM9.

## Introduction

A disintegrins and metalloproteinases (ADAMs) are a family of transmembrane proteins closely related to proteolysis and cell adhesion functions, also known as Metzincins. [1] A total of 40 coding genes have been found in the entire ADAMs family, including 21 human-related genes, and their sequences are similar to the snake venom enzyme integrins family[2]. The research of ADAMs officially began in the 1990s. The researchers found that there is an amino acid sequence with similar functions to snake venom metalloproteinase (SVMP), and began to study it from here [2,3]. The initial stage mainly focused on the gene sequence and protein structure of ADAMs family members. The next stage mainly gathered in the beginning of the 21st century. ADAMs have played a preliminary role in the invasion and adhesion of human diseases, especially malignant tumors [4,5]. At about the same time, preliminary research was conducted on the mechanism of ADAMs in disease. With the advancement of technical methods and the transformation of clinical treatment ideas, in recent years, researchers have gradually carried out research on clinical drugs and specific blockers on the ADAMs family, especially ADAM9, based on known regulatory pathways, [6] and have made great progress.

## Structure of genetic and bioactive material for ADAM9

A disintegrin and metalloproteinase 9 (ADAM9) belongs to type I transmembrane glycoproteins and is an important member of the ADAMs family. The gene is located at 8p 11.23 on the human chromosome, with a full length of 4447BP, including 22 exons. There are currently 10 transcripts for ADAM9, including a defined mRNA (NM_003816.3), three identified lncRNAs (NR_027878.1, NR_027638.1, NR_027639.1) and 6 RNA transcripts which are predicted to progress in the future.

**Figure 1.**
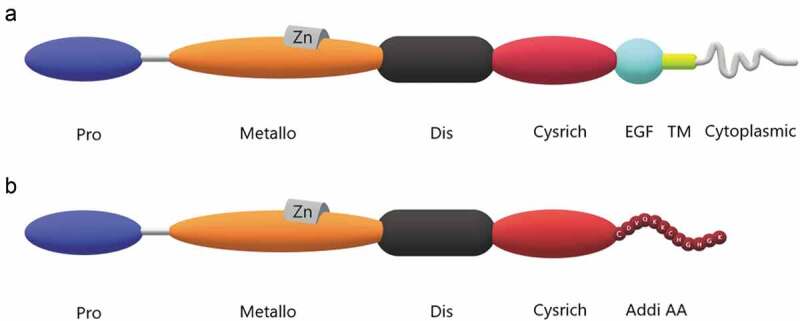
Structure of ADAM9 proteinases. The ADAM9 protein is composed of a few basic domains including propeptide (Pro), metalloproteinase (Metallo), disintegrin (Dis), cystein-rich (Cysrich), EGF-like (EGF), transmembrane (TM), cytoplasmic tail domains and additional amino acids (Addi AA). Proline-rich sequences with special functions at the tail are not marked. A. Protein structure of ADAM9-L. Proline-rich sequences with specific functions at the tail are not indicated. B. Protein structure of ADAM9-S. Additional amino acid species at the end are indicated.

The ADAM9 protein is composed of a leader peptide domain, a metalloproteinase domain, a disintegrin domain, a cysteine-rich domain, an endothelial growth factor-like domain, a transmembrane domain, and an intracellular tail [7–11]. However, analysis of the human genome map in the Celera database revealed the existence of additional predicted ADAM9 transcriptional modalities that could encode a shorter form of ADAM9 that lacks endothelial growth factor-like domains, transmembrane and cytoplasmic domains. Thus, the functional proteins of ADAM9 can be divided into two types; the complete protein is known as ADAM9-L, which is the transmembrane form and it is also the most commonly mentioned. In contrast, the shorter form of the protein is referred to as ADAM9-S and is the secreted form. The immediate cysteine-rich domain, which has an additional 12 amino acids, is lost due to mutation of the group 13 codon to a terminator, causing the loss of the last three regions. (Figure 1). [12,13] ADAM9 as it appears below, unless otherwise specified, refers to ADAM9-L.

The leader peptide domain, as an intramolecular chaperone to ensure the correct folding of its own protein, and has the ability to determine when the protease functions through the cysteine switching mechanism. [7] The metalloprotease domain is one of the domains with distinctive features of ADAMs, followed by the well-known ionizing domain, which can block platelet aggregation and is the area found in snake venoms above. The disintegrin domain also contains a disintegrating element loop, which is composed of 13 amino acid motifs. This is the structural basis for the interaction between the ionizing prime field and integrin. [8] The cysteine-rich domain, endothelial growth factor-like domain and transmembrane domain follow. It is worth mentioning that, in the ADAMs family, ADAM10 and ADAM17 do not contain endothelial growth factor domains. [11] Finally, there is the intracellular tail domain. The length and sequence of this part of the ADAMs family have different changes. ADAMs cytoplasmic domains have been shown to interact with proteins involved in intracellular signal transmission, transport, and cellular structure, and some members contain SH3 domains that can bind Src, and thus can serve as SH3 ligand domain.

**Figure 2.**
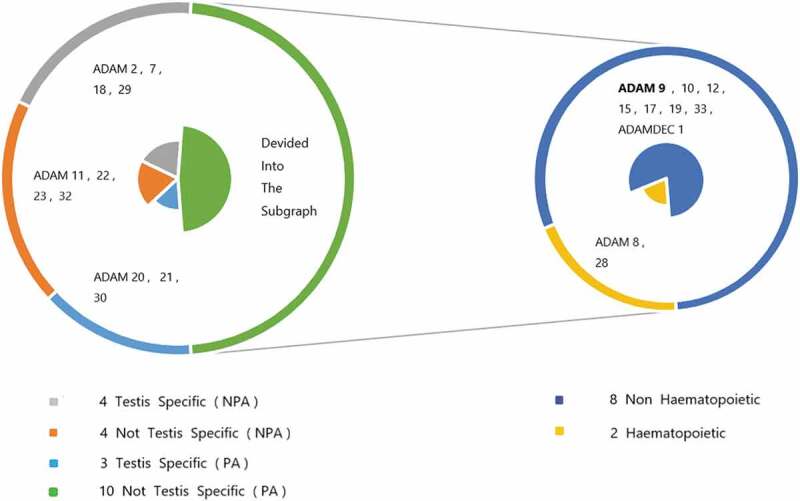
The difference and proportion of protease activity and expression sites caused by the structural characteristics of human ADAMs family members. PA stands for Protease Activity, NPA stands for No Protease Activity.

In the structure of ADAMs, special domains endow them with special protease activity, ability to connect and bind a variety of cells and extracellular matrix-related molecules, which indicates that this enzyme may be functionally capable of controlling the differentiation of tumor cell development stages and the potential for the spread of metastatic cancer. Most of the postulated substrates found so far are transmembrane proteins, so protease activity has become one of the best references to define what functions ADAMs perform in the body. [14] After experimental demonstrations, the degradation of extracellular matrix components and the secretion of growth factors, cytokinins and other substances do require the involvement of active metalloproteases (disintegrin and cysteine-rich regions). In this way, the relevant ADAMs have the ability to regulate cell proliferation and migration. Most members of the ADAMs family are restricted in expressible tissues. The most significant sites are testes and cells with hematopoietic functions, such as ADAM2, ADAM28, etc. Some members, such as ADAM9 and ADAM10, are not subject to this restriction, which means that they can be expressed in multiple tissues and organs and take advantage of the special functions conferred by their own structure to participate in various pathological and physiological processes. Figure 2 shows the relationship between them. Experiments show that ADAMs play a crucial role in various biological processes such as cell adhesion, cell fusion, plasma membrane-associated protein shedding, and intracellular signal transduction. For example, ADAM9, 10, 12 and 17 have protease activity, and ADAM9, 12, 15 and 23 are closely related to the cell adhesion process. [5,15,16] Among these ADAMs protein family members, ADAM9 has been associated with tumorigenesis and metastasis in particular.

In addition, ADAM9 is known to have normal molecular masses of 124 and 84 kDa, respectively. [3,17,18] The intracellular tail of the ADAM9 gene has two proline-rich sequences, namely RPPPPQP and RPAPAPP, and the SH3 domain in the structure gives it a stronger mutual mediating function between proteins. [3] The different domains that make up ADAM9 have mutually independent but complementary functions, giving it distinct characteristics of proteases and adhesion molecules. [1] It is worth noting that the leader domain of the ADAM9 protein can maintain the inactive state of the metalloproteinase domain, and has the ability to activate the cysteine switching mechanism through a complex process, thereby revealing the catalytic site. Studies have found that ADAM9 is highly expressed in a variety of malignant tumors, which plays a role in regulating the occurrence, development, metastasis and invasion of tumors. In addition, it is also involved in inflammation, pathological neovascularization, and allergic reactions. Because ADAM9 has multiple functional domains, it can play corresponding roles in pathological and physiological processes. Its proteolytic activity and the ability to bind to a variety of cellular and extracellular matrix (ECM) related molecules suggest that it may be related to the steps of cancer development and tumor cell metastasis. The active metalloproteinase domain can degrade extracellular matrix components, remove growth factors and cytokines, and thereby regulate physiological functions such as cell proliferation, migration, and angiogenesis. [19–21] Cell adhesion and migration are regulated by the disintegrin domain or cysteine-rich domain. The importance of these areas has been confirmed in different studies.

Tumor metastasis and migration have always been important factors affecting the prognosis of cancer, and high expression of ADAM9 can promote the ability of tumor cell migration and invasion. In recent decades, a lot of research has been carried out on the expression and regulation mechanism of ADAM9 in a series of malignant tumors. The proteolysis of the extracellular matrix and the adhesion of cells to the cell matrix are essential for normal processes such as wound healing and tissue morphogenesis, as well as pathological processes such as tumor cell invasion and metastasis. Various cell surface adhesion proteins and proteases play a very important role in regulating these events. Membrane anchored cell surface adhesion molecule families include cadherins, immunoglobulin superfamily members, integrins, selectins, and syndecan, and membrane anchored cell surface proteases include membrane-type metalloprotease and meprin[22]. ADAM9 is unique among cell surface proteases. It has both a potential adhesion domain and a potential protease domain. Although the domains of these membrane anchored adhesion molecules and proteases are different from each other in sequence, they all perform similar functions. According to the earliest literature reports, ADAM9 is closely related to the domain of SVMPs. SVMPs are encoded by three cDNA classes. N-I encodes pro and metalloproteinase domains, N-II encodes pro, metalloproteinases, and disintegrating protein domains, and N-III encodes pro, metalloproteinases, disintegrating protein-like domains, and cysteine-rich domains, and these structures are very similar to ADAM9.

In addition, the high level of expression of ADAM9 at the edge of multiple tumor metastases and invasion indicates that it is involved in regulating various cancer-related processes, including cancer cell growth, invasion, and cancer grade classification. [23] Unlimited proliferation and tissue changes affected by tissue microenvironment are key features of human cancer. [24] Cancer metastasis is a complex, multi-step process that mainly involves the interaction of cancer cells with components around the extracellular matrix (ECM), including angiogenesis, local invasion, cell migration, intravascular perfusion, extravasation, and secondary growth. The microenvironment of the tumor includes the extracellular matrix surrounding the cells. For most tumors, the effectiveness of the extracellular matrix protein plays a key role in their smooth growth, because these proteins will bind to the cell surface, activate integrin to activate signals to keep cancer cells alive and proliferating. And extracellular matrix is also a rich source of growth factors. Invasion is the first step in cancer cells detaching from the primary tumor and infiltrating the surrounding stomata. This process requires the assistance of matrix proteases. [25]

Matrix proteases, such as urokinase plasminogen activator (uPA), specific matrix metalloproteinases (MMPs) and disintegrins and metalloproteinases (ADAMs), can degrade or reshape the ECM and enable cancer cells to invade locally and eventually form a distant metastasis. Proteases may also promote growth by releasing or activating growth factors such as fibroblast growth factor 2 (FGF-2), growth factor-β (TGF-β), and vascular endothelial growth factor (VEGF), thereby enhancing cell growth, cell migration and angiogenesis capacity. Consistent with their role in promoting tumor cell metastasis, the high level of matrix proteinase expression is also related to the poor prognosis of various malignant tumors. Because of this, proteolytic enzymes have become a key regulator of cancer invasion [26,27].

Like matrix proteases, adhesion molecules also participate in multiple stages of the invasion and metastasis process. At the initial stage of the metastasis pathway, cells must be separated from adjacent cell masses and adhere to the basement membrane. Subsequently, the invasion front undergoes a continuous cycle of adhesion and de-adhesion in order for the invading cells to rely on extracellular matrix (ECM) migration. In the blood circulation, tumor cells can simultaneously attach to blood cells and vascular endothelial cells. Adhesion is mediated by transmembrane proteins such as integrin, cadherin, and selectin. Changes in the expression of these proteins are often found in many malignant tumors.

Among the multiple genes involved in the spread of cancer, the most characteristic are genes encoding matrix-degrading proteases and adhesion proteins. Since both protease and adhesion proteins are involved in the metastasis process, molecules with both protease and adhesion domains may also participate in the metastasis process. After the emergence of continuous research results, our molecular family ADAMs containing these domains (used in the domain of disintegrin and metalloproteinases), especially ADAM9, are closely related to cancer, and are also the hotspot of our focus.

## The main role of Adam9 and its expression regulation mechanism

**Figure 3.**
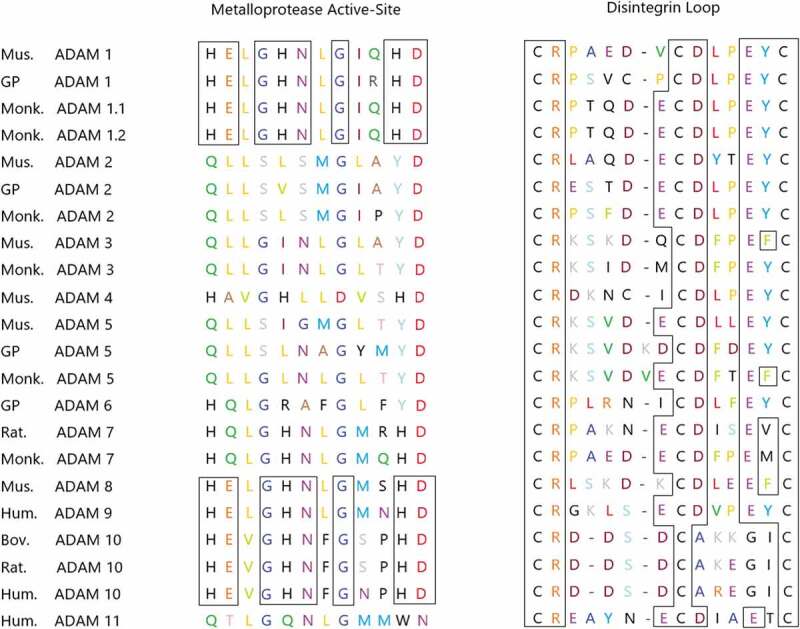
The truncated genes of some ADAMs families expressed in mammals. The residues HEXGHNXGXXHD of the site sequence having the ability to express metalloproteinase activity are shown in the figure. Boxes represent identical residues that are conserved among ADAMs, which encode a consistent sequence of active sites.

According to current research, ADAM9 has been proven to have strong biological activity. On the one hand, as a proteolytically active enzyme, most of its putative substrates are transmembrane proteins, including Pro-HB-EGF, Pro-EGF, Amyloid precursor protein, Kit-ligand, P75 neurotrophic receptor, Insulin B chain, Delta-like ligand I, IGFBP-5, ADAM10, Collagen XVII, Laminin, FGF receptor 2 iiib, etc. [7,17,28–34] It is generally believed that tumor cells can modify the surrounding connective tissue and the metabolism of fibroblasts, thereby synthesizing a matrix that is conducive to the migration of tumor cells.These interactions are partially mediated by cytokines and growth factors released by tumor cells, fibroblasts or inflammatory cells. [35] Only some ADAMs proteins, including ADAM9, have a metalloprotease domain of the catalytic site consensus sequence (HEXGHXXGXXHD), as shown in Figure 3, which indicates that they have catalytic activity, while other proteins do not have this property. [36]

There is currently no in-depth study on the signaling pathways involved in ADAM9 in diseases. The known EGFR signaling pathway process is as follows. ADAM9 activates EGFR by cleaving transmembrane pro-heparin binding epidermal growth factor-like growth factor (pro-HB-EGF) precursors, thereby producing a soluble EGFR ligand, HB-EGF. In turn, HB-EGF binds and activates the intracellular signaling cascade downstream of EGFR, including the AKT pathway, which promoting the growth of normal cells and tumor cells.

In addition to its role in proteolysis, on the other hand, ADAM9 is also involved in cell adhesion. In different types of tumors, ADAM9 interacts with different specific integrins to regulate the occurrence and development of malignant tumors. Currently known integrins that can interact with ADAM9 include α2β1, α6β1, α6β4, α9β1 and αVβ5. [37,38] When ADAM9 interacts with integrin, it can significantly induce the movement of fibroblasts, which can enhance the migration of cells on laminin, one of the components of the basement membrane, thereby achieving the invasion of cancer cells. [4] In summary, the ability of ADAM9 to degrade specific ECM substrates (such as fibronectin), release growth factor stimuli (such as HB-EGF), and cell adhesion functions suggests that this protein may play an important role in cancer progression.

**Figure 4.**
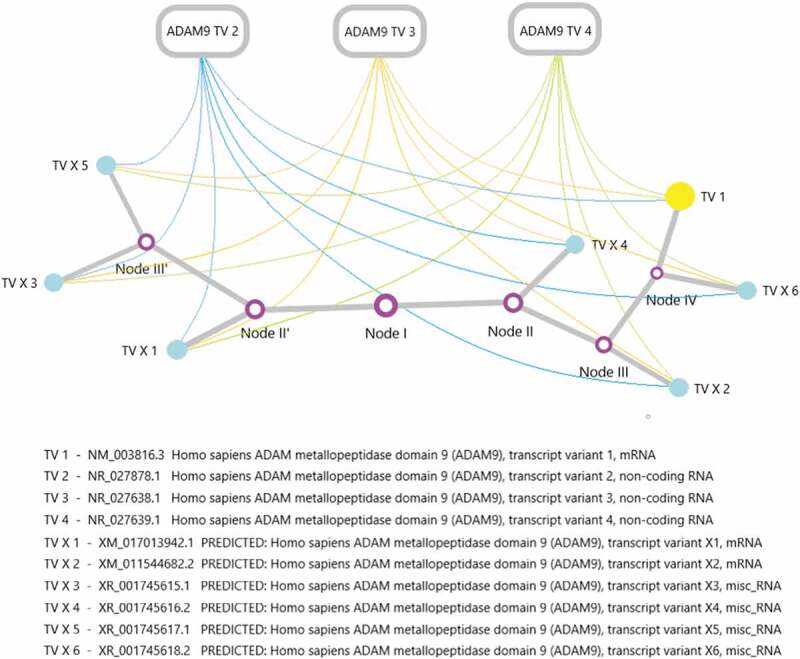
*An evolutionary tree of ADAM9 transcripts and the possible regulatory mechanism exists between the expression of ADAM9 RNA transcripts. The ADAM9 RNA transcript variant evolution tree was generated by maximum likelihood analysis. The abbreviations in the corresponding transcript*s *have been marked, and the nodes in the figure have no practical significance.*

The regulation of ADAM9 activity is a complex process. As mentioned above, ADAM9 contains a total of 10 transcripts, three of which are non-coding RNA (NR_027878.1, NR_027638.1, NR_027639.1). At present, lncRNA is a hot spot in epigenetic research. It is not the noise of genome transcription, and a large number of studies have confirmed that it is essential for the normal growth and differentiation of cells and the occurrence and development of tumors. In recent years, researchers have discovered that the complex and precise regulatory functions played by lncRNA perfectly explain the difficulty of genome complexity. At the same time, people have gradually transitioned from the original model to the dimension of gene expression regulatory network to understand the complexity of life. Therefore, we can reasonably speculate that ADAM9 lncRNA plays an important role in activation and interference in the transcription and expression of ADAM9 effective genes. Figure 4 sketches the specific relationship.

The regulation of ADAM9 activity may be a complex process. At the gene level, two functional proteins have been identified regarding ADAM9, including ADAM9-S and ADAM9-L, due to the transcriptional shear approach. [12,13] Nor are these two proteins simply redundant in their roles; in terms of physiological function, ADAM9-S plays a specific role that overlaps but is not fully related to the intact protein [3,37,39]. In studies on breast cancer, ADAM9-S was found to positively promote tumor cell migration, in contrast to ADAM9-L, which greatly diminished this effect or inhibited tumor migration. Considered in this context, the two ADAM9 splice variants are mutually bound to each other in the organism and act as magical stabilizers[40]. Therefore, ADAM9 can rely on its own genes to alternately express spliced transcription, thereby producing mutant proteins and performing certain functional adjustments. [33] The transcription of ADAM9 RNA is regulated by both position and time, and studies have shown that its splicing can also be regulated. ADAM9 can also be regulated at the protein level. Similar to the SVMP metalloproteinase, ADAM9 metallomatrix protease activity may be regulated by a cysteine conversion mechanism. ADAM9, which encodes a residue of the metalloproteinase active site, contains cysteine in its leader domain. In this mechanism, the cysteine ligand in the leader domain binds to the active site zinc, which can maintain it in inactive state. In addition, the cleavage between proteolytic domains may also regulate some related functions.

MicroRNAs (miRNAs) are a class of small endogenous non-coding RNAs, generally 18–22 nucleotides in length, which target a variety of mRNAs through sequence specificity and play a key role in regulating gene expression after transcription. ADAM9 is one of the direct downstream targets of multiple microRNAs. A single miRNA can down-regulate the expression of multiple target genes, thereby playing a synergistic role in inhibiting or promoting tumor metastasis. Their dysregulation affects cell proliferation, differentiation, invasion and epithelial-mesenchymal transition. [41–43] According to reports, miRNA-126, miRNA-20b and miRNA-33 have a close relationship with ADAM9 in a variety of important tumors. [6,44–49] Therefore, changes in the expression levels of certain miRNAs that are closely related to cancer may be used as markers of tumor progression and lymph node metastasis. [50]

Some evidence suggests that the role of post-translational modification is more important. Some studies have found that ADAM9 can also be activated by the stress regulated by the accumulation of reactive oxygen species (ROS). The elimination of ROS or the use of antioxidants leads to a significant decrease in ADAM9 expression levels. These findings suggest a link between stress-induced signals and ADAM9. In addition to the common mediator ROS, another stress factor, hypoxia, is also a common feature of many solid tumors. Hypoxia can lead to an increase in the expression level of ADAM9 protein, thus affecting the development and progression of various tumors.

In previous studies, the selectivity of the ADAMs family for inhibitors has been confirmed by experiments related to tissue inhibitors of matrix metalloproteinases TIMPs. [51–54] For example, ADAM17 is only inhibited by TIMP-3, and ADAM10 is sensitive to inhibitors TIMP-1 and TIMP-3, but not to TIMP-2/TIMP-4. It is worth noting that the activity of ADAM9 we are discussing here is not regulated by any TIMP. [55]

In addition, ADAM9 can also be affected by drugs and other factors. According to reports, fisetin (3,3 ‘, 4ʹ, 7-tetrahydroxyflavone), which has a certain anti-cancer and anti-invasive ability, is a natural flavonoid widely present in plants. Fisetin can continue to phosphorylate ERK1/2 and reduce the expression levels of ADAM9 protein and mRNA through the ERK1/2 pathway, thereby effectively inhibiting the migration and invasion of related cancer cells. Therefore, based on the related ability of the drug fisetin, it may be a potential drug for the treatment of human cancer. [56]

## ADAM9 involvement in human disease

ADAM9 participates in the regulation of a variety of related tumor processes. Therefore, we have comprehensively refined different previous studies and believe that ADAM9 has played an important regulatory role in the process related to cancer progression, such as promoting cell proliferation and migration, strengthening cell adhesion ability, inhibit cancer cell apoptosis, regulating cell cycle and angiogenesis.

### Breast cancer

Breast cancer is the leading cause of cancer-related deaths among women worldwide. It accounted for 11.6% of new cancer cases in the world in 2018, ranking first with lung cancer. Remote metastasis is the most common cause of death among breast cancer patients. [57,58]For the distribution and potential clinical significance of ADAM9 in breast cancer, it has been involved in a large number of early breast cancer clinical studies. In breast cancer patients, some ADAMs mRNA expression levels were significantly increased in breast cancer surgically removed samples, including ADAM9 mRNA. Compared with normal breast tissue, ADAM9 mRNA is more frequently expressed in breast cancer and fibroadenoma. [59] ADAM9 mRNA is present in approximately 2/3 of primary breast cancers. [60] As in previous studies on MMP-1, MMP-2, MMP-3, MMP-8, MMP-9 and Stromelysin-3, there is no significant correlation between ADAM9 mRNA levels and estrogen receptor levels in breast cancer. [61–64] However, ADAM9 mRNA and progesterone receptor showed a significant negative correlation.

The normal molecular mass of ADAM9 is 124 and 84 kDa.Compared with normal breast tissue, 84 kDa length protein is detected more frequently in primary cancer. [3,17,18] In addition, the relative level of 84 kDa mature protein in primary cancer is significantly higher than that of fibroadenoma, while the relative level of 124 kDa protein is just the opposite, which is a major feature of breast cancer in terms of ADAM9 molecular phenotype. In a large number of breast cancer samples, the expression of ADAM9 protein 84 kDa is higher than that of negative tumors in lymph node positive tumors, and it is positively correlated with HER-2/neu protein levels. [17]Lymph node status, overexpression of HER-2/neu, and occurrence of ductal carcinoma rather than lobular carcinoma all imply that a high level of 84 kDa or mature ADAM9 is associated with poor prognosis of breast cancer. [65–68] That is, compared with normal breast tissue, breast cancer has undergone the above-mentioned differentiation processing or post-translational modification of ADAM9 protein. In short, this high-level form of ADAM9 is most likely related to the poor prognosis of breast cancer patients.

In breast cancer, different miRNA expression profiles including miR-33a and miR-126 have been extensively studied. [69] Compared with adjacent normal tissues, the expression level of miR-126 is often down-regulated in breast cancer specimens. The overexpression of miR-126 significantly reduces the expression level of ADAM9 protein, a key molecule involved in cancer cell metastasis, and thus plays a role in suppressing cell invasion in the development of breast cancer. [70] Microarray analysis by Blenkiron et al. showed that miR-33a is often lost in human primary breast cancer due to chromosomal changes, suggesting that miR-33a may have a tumor suppressive effect in breast cancer. [71] The expression of miR-33a in non-cancerous breast cancer epithelial cells and non-metastatic breast cancer cells is significantly higher than that of high-metastatic breast cancer cell lines, that is, the high level of miR-33a expression can reduce cell proliferation and invasion, significantly inhibit tumor growth and lung metastasis. In addition, the down-regulation of miR-33a in breast cancer tissue is also closely related to lymph node metastasis. MiRNA-33a is a negative regulator of breast cancer cell proliferation and metastasis, but there is no evidence to prove that its downstream action sites include ADAM9. [6] Based on the same regulatory mechanism and effect, we can boldly speculate that miR-33a and miR-126 have the same regulatory signaling pathway, which also affects the proliferation and invasion ability of cancer cells by downstream regulation of ADAM9 expression level.

Some ADAMs may be relevant markers of treatment response. For example, the level of ADAM9 mRNA in tumors is related to the treatment response of the anti-tumor drug tamoxifen, and the high level of ADAM9 protein expression is an important indicator of poor prognosis. [60]

### Esophageal squamous cell carcinoma

Esophageal squamous cell carcinoma (ESCC) is one of the most aggressive malignant tumors of the gastrointestinal tract. [72] Previous studies have reported complex genetic changes and regulatory mechanisms during the progress of ESCC, including ADAM9, which, together with microRNAs (miRNAs), participates in the occurrence development and regulation of ESCC, and forms mutual regulatory loops in many systems. The miRNA analysis based on next-generation sequencing identified 78 miRNAs with different expression levels in ESCC. It was found that microRNA126-3p (miR-126) was silenced by the hypermethylation of its host gene promoter in ESCC tissues, resulting in a significant down-regulation of its expression level, which was associated with the poor prognosis of ESCC. It is worth noting that after high-level expression of miR-126, it can inhibit DNMT1, thereby affecting the expression level of ADAM9 through the pathway, and then regulating the related physiological functions of esophageal squamous cell carcinoma. [73]

ADAM9 is a key target of miR-126 and is regulated by the ‘DNMT1-miR-126 pathway’ in ESCC. ADAM9 is overexpressed in ESCC cell line, and its mRNA level is negatively correlated with miR-126 level. Ectopic expression of miR-126 or silencing of ADAM9 reduces the proliferation and migration of ESCC cells by inhibiting the epidermal growth factor receptor-AKT signaling pathway. [73]

### Lung cancer

In 2018, lung cancer ranked first in the world in terms of morbidity and mortality of malignant tumors, and the mortality rate far exceeded that of other types of tumors, reaching 18.4%[58]. Non-small cell lung cancer (NSCLC) accounts for about 85% of all types of lung cancer, [74,75] and the survival rate of more than 5 years is <15%[76]. Metastatic invasion is the main cause of morbidity and death in patients with NSCLC. After surgical resection of primary lung cancer, it is often accompanied by distant tumor recurrence, which mainly includes lymph nodes, bones and brain. Shintani et al. have found that in NSCLC cells, ADAM9 can regulate the related physiological functions of other adhesion molecules through overexpression and change the sensitivity of surrounding cells to growth factors, thereby enhancing cell adhesion and invasion ability. [77] Liang Chang et al. also reported that the silencing of ADAM9 gene can inhibit the growth of NSCLC in a mouse model. [78]

In lung cancer, the dysregulation of ADAM9 expression has been documented long ago. The high expression of ADAM9 gene in patients with non-small cell lung cancer is closely related to targeted brain metastases. At present, it is known that, compared with human lung cancer squamous carcinoma cell lines that have tropism migration to bone tissue, the mRNA and protein expression levels of ADAM9 are significantly enhanced in strains that have tropism to brain tissue. [77] In addition, the overexpression of ADAM9 in lung cancer cells also leads to an increase in the level of nerve growth factor-induced metastasis, which can lead to larger-scale invasion of brain tissue.

### Colon carcinoma

Colon cancer (CC) is a common tumor that causes a considerable disease burden worldwide. In patients with colon cancer, ADAM9 was found to co-localize with cadherin E, suggesting that it may be more involved in E-cadherin-mediated cancer cell invasion in colon cancer cells. In addition, the ADAM9-S (short) protein, which is one of the two alternative splicing variants of the ADAM9 protein mentioned above, can be converted into a highly aggressive phenotype by binding to α6β4 and α2β1 integrin in noninvasive colon cell lines. [33,79] When ADAM9 is overexpressed in colon cell lines, it can greatly increase the adhesion and migration ability of colon cancer cells.

Chemotherapy resistance is a major obstacle to cancer treatment including colon cancer (CC). The combination of 5-FU and leucovorin is widely regarded as the standard method for the treatment of colon cancer. Although 5-FU can improve the median survival of CC patients for nearly 2 years, the resistance of cells to 5-FU is gradually reducing this effect. In 5-FU resistant tissues and cells, the expression levels of ADAM9 and EGFR were significantly higher than those of 5-FU sensitive tissues and cells, while the expression level of miR-20b was just the opposite. It has been proved that ADAM9 is a direct target of miR-20b. Compared with the corresponding non-cancerous tissues adjacent to cancer, the expression of miR-20b in CC tissue is significantly down-regulated, thereby reducing the resistance of colon cancer cells to 5-FU. In addition, miRNA-20b can also directly target ADAM9 and inhibit ADAM9/EGFR gene expression, thereby delaying the cell cycle progression of CC cells in the G1/S phase, inhibiting cell proliferation and inducing apoptosis[80].

### Bladder cancer

Bladder cancer is one of the most common malignant tumors of the genitourinary system. It is mainly characterized by metastatic cell carcinoma that has a higher degree of deterioration and is prone to infiltration and metastasis. For patients with metastatic bladder cancer who are not viable for resection, even after systemic chemotherapy, the median survival time is still only 7–20 months, and the 5-year survival rate is only 5.5%[81]. Cancer cells invade and metastasize normal muscles, which is the main cause of death of patients with bladder cancer. Some researchers have found that in bladder cancer tumor tissues and normal tissues adjacent to the cancer, the expression of ADAM9 is increased, and the degree of tumor deterioration is proportional to its expression level. That is, the expression level of ADAM9 can be used as a sign of poor prognosis in bladder cancer[82].

In bladder cancer, the expression levels of miRNA-126 and ADAM9 showed a significant negative correlation. The specific regulatory mechanism is that miR-126 directly acts on ADAM9 through negatively regulated expression, and further regulates the invasion of bladder cancer cells by affecting its expression level[82].

### Renal cell carcinoma

Renal cell carcinoma (RCC) is one of the deadliest malignant tumors of the urinary system, accounting for 2–3% of global cancer patients. Lymph node invasion, vascular invasion and tumor cell metastasis are important prognostic factors affecting renal cell carcinoma. Compared with adjacent normal tissues, ADAM9 mRNA levels are significantly up-regulated in renal cell carcinoma. At the protein level, it has a significant relationship with higher tumor development grade, positive lymph node status, and distant metastasis. In addition, in the experimental analysis, it was also found that ADAM9 protein expression was significantly related to the shortening of patient survival[83].

For the treatment of renal cell carcinoma, in the presence of cancer cell metastasis, in addition to nephrectomy, the use of immunotherapy drugs is needed to prolong the median survival time of the patient. Fisetin is a naturally-occurring flavonoid compound with anti-tumor effects such as anti-proliferation, anti-oxidation, anti-angiogenesis, and the ability to promote cell cycle arrest and apoptosis. As a drug, fisetin can inhibit the cell proliferation and colony-forming ability of renal cell carcinoma cell lines, and can play a blocking role in the proliferation of renal cancer cells, so that the cell cycle is arrested in the G2/M phase. [84] In the process of fisetin regulating the expression levels of ADAM9 and CTSS and exerting tumor suppressing effects, there are various conduction pathways including MAPK signaling pathway and ERK pathway. Fisetin exerts its cytotoxic effect by inhibiting or activating the MAPK signaling pathway and regulating its pathway. ERK signaling pathway can be activated by fisetin, which in turn down-regulates the expression levels of CTSS and ADAM9, thereby inhibiting cell proliferation, invasion and migration, and forming an anti-tumor metastasis effect. [85]

### Prostate cancer

The underlying mechanism of ADAM9 in prostate cancer has also been discovered: ADAM9 can cleave and release epidermal growth factor and FGFR2iiib from cells, both of which play a key role in the pathogenesis of prostate cancer. [34] Peduto et al. reported that ADAM9 can hydrolyze and release the ligands of epidermal growth factor receptor and fibroblast growth factor receptor on the surface of the target exfoliated cells to promote the proliferation of prostate cancer cells. Fisher et al. shows that the oxidation and osmotic pressure on tumor cells will increase the shedding of heparin-binding epidermal growth factor (EGF), and this process is caused by the proteins of the ADAM family (specifically ADAM9, ADAM10 and ADAM17) Processed. In addition, prostate cancer has androgen-dependent properties in the early stages of development, but with the growth of cancer cells, it can be transformed into non-androgen-dependent tumors. [86,87] During this transition, microarray analysis can detect that the expression level of ADAM9 protein in human malignant prostate tissue is significantly higher than that of benign prostate tissue. [88] Under the same conditions, treatment of male hormone-dependent prostate cancer cells with dihydrotestosterone (DHT) also showed the results of upregulation of ADAM9 mRNA expression.

In the process of prostate cancer, ADAM9 and ROS are accompanied, which may involve a kind of stress. ROS can induce the expression of ADAMs by activating p38 mitogen protein kinase. The induced ADAM protein is responsible for the hydrolysis and release of heparin-bound EGF, which further promotes cancer cell growth and survival through an EGFR-dependent mechanism. Studies have shown that when human prostate cancer cells are exposed to stress conditions such as cell crowding, hypoxia and hydrogen peroxide, the expression levels of ADAM9 mRNA and protein also increase. In conclusion, intracellular ROS/hydrogen peroxide produced by cellular stress also participates in the regulation of ADAM9 expression. For prostate cancer cells, ADAM9 is likely to be responsible for supporting its survival and tumor progression. Because the expression of ADAM9 is reduced, prostate cancer cells will undergo apoptosis. [88]

In summary, these results reveal that ADAM9 is involved in the possible pathogenesis of prostate cancer, and also indicate that ADAM9 is likely to become a good target for anti-tumor drugs in the future. Studies have found that overexpression of ADAM9 in the mouse prostate can lead to abnormal acinar epithelium and high-grade prostate intraepithelial neoplasia. [34] Strictly speaking, inducing normal cells to become cancerous requires more experiments to support the theory and mechanism, but it also illustrates the close relationship between ADAM9 and cancer from another angle.

### Brain tumors

Glioblastoma is a type of intracranial tumor that accounts for 3% −8% of all cancer-related deaths. Research on malignant gliomas has made considerable progress, but to a large extent it is still untreated, and the median survival time of high-grade gliomas is no more than 15 months. One of the main reasons for the poor prognosis of gliomas is their aggressiveness in the central nervous system, with a high probability of generating multiple metastatic growth lesions.The few cell populations with the ability to proliferate and self-renew are considered to be the source of tumor growth that maintains malignant gliomas, which automatically produce more differentiated next-generation gliomas. These self-renewing and transforming precursors, known as glioma stem cells or brain tumor initiating cells (BTICs), are believed to play an important mediating role in the treatment of their invasion and recurrence of multiple foci. [89–91] The expression levels of ADAM9 gene were significantly increased in different BTIC lines.

The microenvironment of the tumor includes the ECM surrounding the cells. For most tumor types, the availability of ECM proteins is a key factor for growth.The invasiveness of gliomas is mediated by the interaction between glioma cells and extracellular matrix (ECM), and formed by the proteolytic cleavage of ECM protein by proteases secreted by tumor cells. ECMs of malignant gliomas include vitronectin, proteoglycan, collagen I and IV, osteopontin and tenascin C (TNC). Among them, TNC is the most significant glioma ECM component, and its expression level is closely related to the grade of glioma development. Studies have found that in the three-dimensional matrix of type I collagen, TNC can interact with ADAM9 by using pathways including JNK signaling pathway, thereby stimulating the invasiveness of BTICs. Glioma cells on the margin of tumor progression can produce TNC, which then stimulates BTIC aggressiveness in a metalloproteinase-dependent manner. [92,93] After screening global gene expression, ADAM9 is a potential regulatory factor for TNC to stimulate BTIC invasion.

In addition, after knocking out ADAM9 with siRNA, it was found that the proteolytic activity of the tumor disappeared, supporting ADAM9’s advantages over related proteases. MiRNA-33a can promote the self-renewal of glioma-initiating cells. [94] The internal mechanism has been speculated before, and there is a high probability that it can be assumed to play a role by adjusting the level of ADAM9. Taken together, these results identify an important molecule ADAM9 in regulating BTIC invasiveness and maintaining tumor cell regeneration.

### Gastric carcinoma

Gastric cancer (GC) is the second leading cause of cancer-related deaths worldwide in 2018. [58] After diagnosis of advanced gastric cancer, the median survival time is only about one year. After diagnosis of advanced gastric cancer, the median survival time is only about one year, and its poor prognosis is mainly due to the early gastric cancer cell invasion and high metastatic activity. Therefore, advanced gastric cancer is one of the most aggressive tumors in gastrointestinal malignant tumors. In vivo experiments on gastric cancer, especially the highest level of ADAM9 overexpression at infiltrating border cells bordering non-cancerous epithelial cells, which are mainly distributed in the cell membrane and cytoplasm, which leads to a decrease in the invasion rate by inhibiting the EGFR/ERK signaling pathway. [95,96] In gastric cancer cells, its protease activity is moderately correlated with the expression level of ADAM9 protein (128kDa), but it is significantly improved with the expression level of ADAM9’s smaller molecular weight (84kDa). When ADAM9 was knocked out or the specific targeting monoclonal antibody (RAV-18) was used, gastric cancer cell proliferation and invasion could be inhibited in cells that were originally at high levels of ADAM9 expression. [97]

In addition, hypoxic stress (1% oxygen) is a feature of many solid tumors, including gastric cancer. It increases the expression and activity of ADAM9 and increases the invasion of the tumor, which may help the growth and spread of gastric cancer. In addition to regulating the occurrence of gastric cancer, ADAM9 protein also plays an important role in promoting intra-abdominal metastasis and diffusion[97].

### Hepatocellular carcinoma

Hepatocellular carcinoma (HCC) is a high-risk malignant tumor in the world. After liver injury occurs, activated hepatic stellate cells (HSCs) participate in the inflammatory response by secreting MMPs, leading to increased ECM remodeling and increased matrix deposition. During this process and the invasion stage of hepatocellular carcinoma, ADAM9 promotes the invasion of HCC by degrading the basement membrane components including laminin-1. [33] In addition, ADAM9 is expressed differently in liver stromal cells and epithelial cells. [98] The specific regulatory role and regulatory mechanism that ADAM9 plays in the development of HCC cells need to be further studied.

### Melanoma

In melanoma tissues, ADAM9 protein is strongly expressed in tumor cells of the surrounding dermis and stromal cells adjacent to the leading edge of the tumor. In contrast, ADAM9 mRNA cannot be detected in normal skin, meaning that the expression of ADAM9 in melanoma and stromal cells may be controlled by changes in related cells during tumor progression. [60,99,100] Matrix proteolysis is achieved by different types of proteases, and many proteases reported to function are induced and activated by cell-matrix interactions in melanoma cells. [101–104]

The strong expression of ADAM9 in the melanoma anterior margin and stromal cells near the tumor indicates that the tumor-stromal interaction promotes the induction of ADAM9 in vivo. [99] This also means that the expression of the ADAM9 gene must not be the active result of cancer cells, but can also occur in the induction of environmental factors. Collagen type I is a key factor for cell-matrix interaction to regulate ADAM9.

Several studies have shown that during the transformation of benign melanocytes into melanoma cells, the expression of various cell-cell and cell-matrix receptor proteins has changed. [105] No evidence ofinteractions between isotype cells was found in ADAM9 regulation, but this does not exclude interactions between tumor cells and atypical cells such as stromal fibroblasts, inflammatory cells or endothelial cells, which may play an important role in ADAM9 expression.

### Pancreatic carcinoma

Pancreatic cancer is a malignant tumor of the digestive tract with a high degree of malignancy, and there are great difficulties in its diagnosis and treatment. It has the characteristics of early invasion of surrounding tissues and rapid spread to distant organs. Its prognosis is extremely poor, and its 5-year survival rate is basically <1%. Experiments have reported that through the analysis of mRNA expression levels in micro-dissected pancreatic cancer specimens, ADAM9 overexpression was found in pancreatic tumor cells. [106] Hamada et al. demonstrated that the expression of miR-126 and the knockout of ADAM9 gene in pancreatic cancer cells both lead to enhanced cell migration and invasion capabilities. Combined with the downstream regulation mechanism of miR-126 I explained above, ADAM9 is an indispensable part. [48] In conclusion, the miR-126/ADAM9 axis plays an important role in inhibiting the invasive growth of pancreatic cancer cells.

## Other diseases

In addition to having a close relationship with tumors, ADAM9 has also been reported in other diseases. For example, in the study of Alzheimer’s disease, ADAM9 was found to play a certain role in the pathogenesis. [107] In terms of cardiovascular, ADAM9 has also been found to work with ADAM15 to regulate integrin αvβ3 and α5β1, thereby affecting the progress of atherosclerosis. [108] These may be the marginal functions of the ADAM9 protein and can be used as part of its comprehensive understanding.

Because of the similar structural domain to ADAM9, other members of the ADAMs family also play their roles in the disease. According to reports, ADAM8, 10, 12, 15, 29 and other members also play a role in cancer. [109–113] In addition, rheumatoid arthritis, Down’s syndrome and other diseases have also been reported in few cases with members of the ADAMs family [114–116].

## Discussion

The main features of malignant tumors are their ability to invade surrounding tissues, enter blood vessels and lymphatic systems, and spread to distant organs through metastasis. In Europe and the United States, cancer remains the second leading cause of death. [117–119] The aggressiveness of malignant tumors is an important cause of poor tumor prognosis. Therefore, it is a reasonable goal to improve the therapy to recognize the mechanism of tumor invasion and specifically block or regulate some of these links. The movement of cells requires the participation of the internal mechanism of the cell, the external mechanism of the cell and environmental factors. The integrin on the cell surface binds to the ECM protein to form a focal adhesion complex at the contact point, which can help regulate the actin cytoskeleton. At the same time, related proteases are also secreted. These proteases can help transform the extracellular environment and allow the cells to advance. Many protease families are involved in the invasion process, including metalloproteinases MMPs and ADAMs [120,121].

ADAMs are membrane-anchored enzymes that play a key role in the proteolysis of many membrane-bound molecules (including growth factors, receptors, and adhesion molecules). These molecules affect a variety of cellular processes, including cell proliferation, differentiation, invasion and migration. [122] When the ADAM9 gene was knocked out, the mice showed vitality and fertility, and showed no pathological changes. [123] Compared with other ADAMs genes, the knockout of the ADAM9 genedoes not show common important characteristics of male infertility, sperm migration and adhesion defects, [124–127] which shows that the importance of the ADAM9 gene is more focused on regulating the occurrence and development of tumors. In this review, we show recent research data suggesting that changes in ADAM9 expression have been found in different tumor types. This indicates that these proteins are involved in different steps of cancer progression, including the carcinogenesis caused by regulating the proliferation and invasiveness of tumor cells.

**Figure 5.**
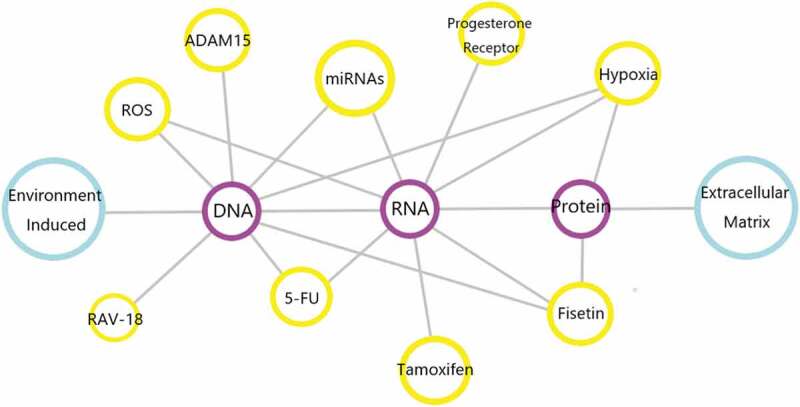
Genes or biological components involved in the regulation of ADAM9. In human diseases and related malignant tumors, the above chart shows the genes and biologically active components that can regulate ADAM9. Connected by gray lines indicates that the two can interact, and the pathways involved are not marked.

In the body, there are quite a few factors that play their roles in regulating ADAM9 gene expression. The specific factors have been summarized in Figure 5. With the progress of research, the exact mechanism of these proteases in the occurrence or development of diseases will be gradually discovered. It is worth mentioning that these proteases may become useful tools and can be used clinically as biomarkers for early cancer diagnosis or late tumor prognosis. However, since there are more than 30 similar proteases (including MMPs and ADAMs) in humans, the design of specific ADAM9 preparations, such as the design of siRNAs or monoclonal antibodies, is a real challenge. As we have already mentioned above, ADAM9 has its properties, its mutant spliceosome constitutes a certain degree of steady state in the organism and it is expressed in monocytes, activated macrophages, fibroblasts, epithelial cells, activated vascular smooth muscle cells and keratinocytes, [17,108,128–131] and if ADAM9 is completely blocked it will also have some negative effects on normal cellular and physiological conditions. Therefore, for it the agent of action will be different from other targets and the mechanism may be simple or more complex. The answer to this question requires further exploration in the future.

Since the discovery of the first batch of sperm-related proteins in the early 1990s, our understanding of the mammalian ADAMs family has made considerable progress. [132] To do a good job, one must first sharpen the tools. After fully understanding therelated functions of ADAM9 in cancer, the next step should focus on how to specifically regulate it. Therefore, future work should be concentrated in the following aspects. The first point is to further explore the unknown potential role of ADAM9 in malignant tumors, especially cancer invasion and metastasis. The second point is to further clarify the regulatory mechanism of ADAM9 and other ADAMs family members related to tumors. The third point is to continue to investigate in depth how to appropriately modulate the orientation of the two splice variants of ADAM9 to constitute a steady state, or to partially block ADAM9, by specific agents or special methods. At a dosage or in a manner that is acceptable for normal tissue harm effects, the ability to reduce the metastatic invasiveness of the above related cancers is reduced and the survival time of patients is prolonged.
